# Responses of *Synechocystis* sp. PCC 6803 to heterologous biosynthetic pathways

**DOI:** 10.1186/s12934-017-0757-y

**Published:** 2017-08-15

**Authors:** Konstantinos Vavitsas, Emil Østergaard Rue, Lára Kristín Stefánsdóttir, Thiyagarajan Gnanasekaran, Andreas Blennow, Christoph Crocoll, Steinn Gudmundsson, Poul Erik Jensen

**Affiliations:** 1 Copenhagen Plant Science Centre, Department of Plant and Environmental Sciences, University of Copenhagen, Thorvaldsensvej 40, 1871 Frederiksberg C, Denmark; 2 Center for Systems Biology, University of Iceland, Sturlugata 8, 101 Reykjavik, Iceland; 3 ISBP-INSA de Toulouse, Avenue de Rangueil, 31077 Toulouse, France

**Keywords:** Cyanobacteria, Metabolism, Terpenoids, Amino acids, Metabolic modelling

## Abstract

**Background:**

There are an increasing number of studies regarding genetic manipulation of cyanobacteria to produce commercially interesting compounds. The majority of these works study the expression and optimization of a selected heterologous pathway, largely ignoring the wholeness and complexity of cellular metabolism. Regulation and response mechanisms are largely unknown, and even the metabolic pathways themselves are not fully elucidated. This poses a clear limitation in exploiting the rich biosynthetic potential of cyanobacteria.

**Results:**

In this work, we focused on the production of two different compounds, the cyanogenic glucoside dhurrin and the diterpenoid 13*R*-manoyl oxide in *Synechocystis* PCC 6803. We used genome-scale metabolic modelling to study fluxes in individual reactions and pathways, and we determined the concentrations of key metabolites, such as amino acids, carotenoids, and chlorophylls. This allowed us to identify metabolic crosstalk between the native and the introduced metabolic pathways. Most results and simulations highlight the metabolic robustness of cyanobacteria, suggesting that the host organism tends to keep metabolic fluxes and metabolite concentrations steady, counteracting the effects of the heterologous pathway. However, the amino acid concentrations of the dhurrin-producing strain show an unexpected profile, where the perturbation levels were high in seemingly unrelated metabolites.

**Conclusions:**

There is a wealth of information that can be derived by combining targeted metabolite identification and computer modelling as a frame of understanding. Here we present an example of how strain engineering approaches can be coupled to ‘traditional’ metabolic engineering with systems biology, resulting in novel and more efficient manipulation strategies.

**Electronic supplementary material:**

The online version of this article (doi:10.1186/s12934-017-0757-y) contains supplementary material, which is available to authorized users.

## Background

Metabolic engineering is the manipulation of living organisms to produce a desired product. This manipulation commonly refers to the alteration of the metabolism and the strain optimization required to increase production titers. Most of the metabolic engineering breakthroughs have taken place using heterotrophic model organisms, such as *Escherichia coli* and *Saccharomyces cerevisiae*; however, this limited choice of organisms restricts the application potentials, thus highlighting the need of expanding towards other organisms [1]. Cyanobacteria are promising biotechnological organisms that combine the capability to perform photosynthesis like higher plants, with simple, unicellular organization like *E. coli* and *S. cerevisiae*. The biosynthetic and metabolic properties of cyanobacteria, together with their facile genetic manipulation, allow for the production of various industrially interesting compounds [2–4].

Amino acids are among the building blocks of life, but they also have tremendous commercial importance, both as end-products and as precursor molecules for production of various complex biomolecules [5]. Aromatic amino acids, and their precursors in particular, are the starting points of many natural compounds, including flavonoids, glucosinolates, and lignins [2, 6]. Terpenoids are another diverse class of metabolic products. They consist of a carbon skeleton that is formed from one or multiple isoprenoid units, further decorated by modifying enzymes [7]. Their applications range from biofuels to anticancer and anti-malarial drugs. Isoprene has been produced in cyanobacteria in the g/L of culture titer range [8, 9], and the biosynthetic pathway of the diterpenoid 13*R*-manoyl oxide—the precursor of the cAMP activator forskolin—was expressed in *Synechocystis* PCC 6803 (hereafter *Synechocystis*) [10]. More recently, we showed that *Synechocystis* can produce dhurrin, a cyanogenic glucoside, in a light-dependent manner, using tyrosine and UDP-glucose as precursor molecules [11].

At present, the product titers of most commercially interesting products obtained in cyanobacteria are low [2]. This may reflect a lack of knowledge on the effects of introducing foreign pathways in the host organisms. In this work, we focused on the production of dhurrin and on 13*R*-manoyl oxide in *Synechocystis*. The strategy was to employ genome-scale modelling to determine metabolic relationships and we experimentally determined the levels of key metabolites, with the aim to uncover responses that affect the optimal production of amino acid and isoprene derived compounds. Our data reveal specific metabolic crosstalk with the inserted pathways and provide important information on the mechanisms *Synechocystis* employs to regulate its metabolism.

## Methods

### Strains, growth conditions and cell harvesting

The *Synechocystis* strains used in this work are listed in Table 1. Cyanobacterial cultures were grown in 50 mL glass tubes (culture volume 20 mL), in BG-11 media at 30 °C, continuously supplied with 3% CO_2_ enriched air and supplemented with 50 µg/mL spectinomycin. The light intensity was steady at 50 μmol photons/s/m^2^. Cultures were grown for 24 h and then induced with 2 mM isopropyl β-d-1-thiogalactopyranoside (IPTG). Cells were harvested after 48 h of growth by centrifugation at 14,000*g* for 2 min. The cell pellets were flash-frozen in liquid nitrogen and stored at −80 °C until further use.

**Table 1:** *Synechocystis* PCC 6803 strains used in this work

Strain	Information	Reference
Control	Empty pDF-trc vector/spec^R^	[12]
DH	Codon-optimized dhurrin biosynthetic pathway in an operon (*CYP79A1*, *CYP71E1*, and *UGT85B1*) in pDF-trc vector/spec^R^	[11]
MO	Codon-optimized 13R-manoyl oxide biosynthetic pathway in an operon (*CfTPS2* and *CfTPS3*) in pDF-trc vector/spec^R^	This study

### Genetic modification of *Synechocystis*

*CfTPS2* [GenBank: KF444507] and *CfTPS3* [GenBank: KF444508], encoding the diterpene synthases that convert geranylgeranyl diphosphate (GGPP) to 13*R*-manoyl oxide [13], were codon-optimized using the OptimumGene™ optimization tool for *Synechocystis*, available from GenScript and inserted into the pDF-trc shuttle vector [12]. Vector construction and *Synechocystis* transformation were performed as described in [11]. Briefly, the two genes, each preceded by a ribosome binding site, were inserted as an operon into a linearized pDF-trc vector using T4 DNA ligase (New England BioLabs). *Synechocystis* was transformed via triparental mating [14]. The construction of the dhurrin strain was previously reported [11].

### Identification and quantification of 13*R*-manoyl oxide

Cell pellets were homogenized in a Bullet Blender^®^ homogenizer (Next Advance Inc.) in methanol using 0.15 mm zirconium oxide beads. This was followed by a hexane extraction for 3 h at room temperature at the Roto-Shake Genie^®^ (Scientific Industries Inc.) revolving at 25 rpm. The presence of 13*R*-manoyl oxide in the extract was confirmed by gas chromatography–mass spectrometry (GC–MS) analysis using a GCMS-QP2010 Ultra (Shimadzu), and the method are described in [10]. Manoyl oxide was quantified using GC-FID in a SCION 436 GC-FID (Bruker) as described in [10], with the following modifications: 2 μL sample was injected, and the amount of manoyl oxide was quantified via a standard curve.

### Pigment analysis

The photosynthetic pigments were extracted from cell pellets in 90% (v/v) methanol and analyzed by high performance liquid chromatography (HPLC) in a LC-20AT High Pressure Liquid Chromatographer with a SPD-M20A Photodiode Array Detector (Shimadzu, Japan) as described in [15]. Pigments were identified by comparing retention times and absorption spectra with standard pigments (DHI, Hørsholm, Denmark), and their concentration determined via external authentic standards.

### LC–MS analysis for identification of dhurrin and amino acids

Cell pellets were homogenized as described in “Identification and quantification of 13*R*-manoyl oxide” section in 80% (v/v) methanol. Extracts were diluted to 20% (v/v) methanol concentration and filtered using 0.22 µm Ultrafree-MC GV Centrifugal Filter (Merck Millipore). The dhurrin analysis was performed as described in [11].

The amino acid analysis was performed as described in [16], adopted from a protocol by [17] originally described for aspartate-derived amino acids by [18]. Briefly, samples for amino acid quantification were spiked with ^13^C, ^15^N-labeled amino acids (Algal amino acids, Isotec, Miamisburg, USA) at a concentration of 1 µg/mL prior to analysis by LC–MS.

Chromatography was performed using an Advance UHPLC system (Bruker, Bremen, Germany). Separation was achieved using a Zorbax Eclipse XDB-C18 column (50 × 4.6 mm, 1.8 µm, Agilent Technologies, Germany). Formic acid (0.05%) in water and acetonitrile (supplied with 0.05% formic acid) were employed as mobile phases A and B, respectively. The elution profile was: 0–1.5 min, 3% B in A; 1.5–3.1 min, 3–100% B in A; 3.1–3.6 min 100% B, 3.6–3.9 min 100–3% B in A and 3.9–5.0 min in 3% B. The mobile phase flow rate was 500 µL/min. The column temperature was maintained at 40 °C. The chromatography system was coupled to an EVOQ Elite TripleQuad mass spectrometer (Bruker, Bremen, Germany) equipped with an electrospray ion source operated in positive ionization mode. The instrument parameters were optimized by infusion experiments with pure standards (Amino acid standard mix, Fluka, St. Louis, USA). The ion spray voltage was maintained at 3000 V. Cone temperature was set to 300 °C and cone gas to 20 psi. Heated probe temperature was set to 300 °C and probe gas flow set to 50 psi. Nebulizing gas was set to 60 psi and collision gas to 1.6 mTorr. Multiple reaction monitoring was used to monitor parent analyte ion → product ion transitions. Multiple reaction monitoring and corresponding collision energies can be found in [16]. Both Q1 and Q3 quadrupoles were maintained at unit resolution. Bruker MS Workstation software (Version 8.1.2, Bruker, Bremen, Germany) was used for data acquisition and processing.

### Glycogen quantification

Glycogen concentration was determined by enzymatic hydrolysis of the cellular glycogen polysaccharide and quantifying the derived glucose. Pelleted cells were homogenized in a Bullet Blender^®^ homogenizer (Next Advance Inc.). The broken cells were resuspended in a [10 mM K_2_PO_4_, 1 mM CaCl_2_, 0.02% w/v NaN_3_] buffer, preheated at 80 °C. The samples were incubated for 20 min at 80 °C with the addition of 30 U thermostable α-amylase (Megazyme), and were subsequently incubated overnight with the addition of 7 U pullulanase M1 and amyloglucosidase (Megazyme). The extracts were spun down, the supernatant filtered, and analyzed by High Pressure Anion-Exchange Chromatography using a Pulsed Amperometric Detection (HPAEC-PAD) BioLC system (Dionex, Sunnyvale, CA, USA) equipped with an AS50 autosampler, GS50 gradient pump and ED50 electrochemical detector. Samples (10 μL) were injected and separation of glucose from other sample components was performed using a CarboPac™ SA10 column with a flow rate of 0.35 mL/min and isocratic 1 mM NaOH as eluent. Peaks were evaluated using Chromeleon V.6.70 and glucose concentrations calculated by external authentic glucose standards.

### Oxygen evolution

Cyanobacterial samples were collected 48 h after inoculation. Samples were diluted with fresh BG11 media to a concentration of ~4 μg chlorophyll/mL and dark-adapted for 5 min. Prior to measurements, NaHCO_3_ was added to the samples to a final concentration of 10 mM. Oxygen evolution was measured using a Clark-type electrode (LD2/3 Electrode chamber, Oxy-Lab Systems, Hansatech Instruments^®^). Oxygen evolution was measured at increasing light intensities (23, 55, 115, 420, 812 and 1660 µmol/m^2^/s) provided with a Schott lamp (HLX 64634 EFR 150 W Osram^®^ Xenophot) with a 0.6 neutral density optic filter.

### Metabolic modelling

The iJN678 metabolic network reconstruction of *Synechocystis* [19] version 1.1 was used to study the effects of expressing the heterologous pathways in silico. The model was updated to include the Entner–Doudoroff pathway [20], modifications in the tyrosine biosynthesis from arogenate according to [21] and corrections from [22]. The reactions involved in the heterologous pathways and the model updates are listed in Additional file 1: Table S1. As a result, three networks were obtained, one corresponding to the wild type which was used as a control, one corresponding to the dhurrin strain, and one to the manoyl oxide strain. The models were analyzed by randomly sampling feasible flux values from the networks [23, 24]. Prior to random sampling, the metabolic networks were constrained with the experimental growth and product formation rates (lower bounds only, 2.3 μmol dhurrin/gDW/h and 0.8 μmol manoyl oxide/gDW/h respectively). The complete set of model constraints is given in Additional file 1: Table S5 and the model is provided in SBML format in Additional file 3. Random sampling is an unbiased method in the sense that a biological objective (e.g. maximization of biomass) is not needed. The method results in a number of flux vectors representing feasible states of the network where the elements of a single flux vector correspond to fluxes in individual reactions. The mean and standard deviation of flux values were computed to enable comparison of fluxes in the heterologous pathways versus a control. For the models corresponding to the engineered strains, pairwise correlation coefficients were computed between all reactions in the network to enable identification of network reactions most strongly correlated with product formation [24]. The computational analysis was carried out in the Matlab environment (Mathworks) using the COBRA toolbox version 2.0 [25] and the Gurobi solver.

## Results

### Expression of the two heterologous pathways

**Fig. 1:**
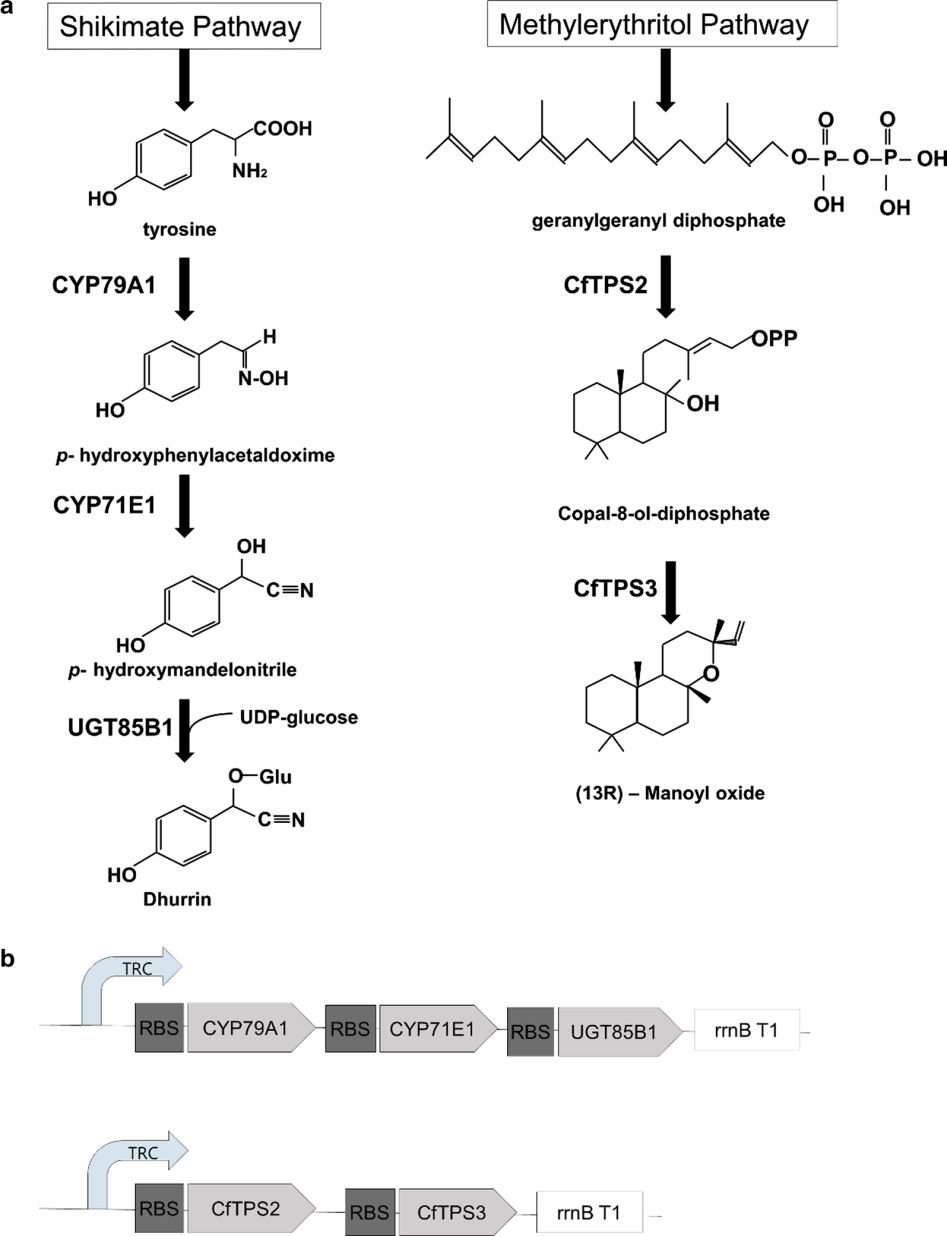
Dhurrin and 13*R*-manoyl oxide biosynthetic pathways and the genetic constructs for their expression in *Synechocystis*. **a** The precursors tyrosine and geranylgeranyl diphosphate are derived from the shikimate and the methylerythritol pathway respectively. The final glycosylation step in dhurrin biosynthesis connects the cyanogenic glucoside formation with the sugar metabolism. **b** The genes encoding enzymes of the dhurrin and the manoyl oxide biosynthetic pathways, respectively, were arranged in operons, under the control of the trc promoter (TRC). The ribosome binding site (RBS) ribo* [39] is incorporated before the 5′ of each gene, and each operon concludes with the rrnB terminator (rrnB T1). Both linear constructs were inserted in the pDF-trc expression vector

Dhurrin is a cyanogenic glucoside derived from tyrosine, and its biosynthetic pathway involves two cytochrome P450s that convert tyrosine into *p*-hydroxymandelonitrile and a UDP-glycosyltransferase that transfers a glucose from UDP-glucose (Fig. 1a). Dhurrin was produced in *Synechocystis* by inserting the three biosynthetic enzymes as an operon into the pDF-trc shuttle vector [11] (Fig. 1b). The second compound we chose is the forskolin precursor 13*R*-manoyl oxide, a diterpenoid derived from geranylgeranyl diphosphate by the enzymatic action of two diterpene synthases [13] (Fig. 1a). *Synechocystis* was previously engineered to produce manoyl oxide, where the diterpene synthases were incorporated in a genomic neutral site, under the transcriptional control of a *psbA* (aconstitutive promoter) and a nickel-inducible promoter [10]. In order to directly compare the effects of the two heterologous biosynthetic pathways, we expressed the manoyl oxide pathway in a way similar to the dhurrin one: we codon-optimized the two diterpene synthases and inserted them into the pDF-trc vector, thus expressing the two genes under the strong, IPTG-inducible trc promoter (Fig. 1b).

**Fig. 2:**
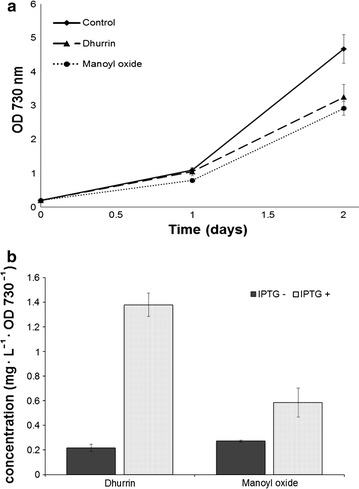
Growth rates and production titers of the two cyanobacterial strains, producing dhurrin and 13*R*-manoyl oxide. The *error bars* represent the standard deviation within three biological replicates. **a** Initial growth rate is represented as a function of the optical density at 730 nm. Cells were grown in air supplemented with 3% CO_2_, and were induced with 2 mM IPTG 24 h after inoculation. **b** Dhurrin (DH) and 13*R*-manoyl oxide (MO) accumulation at 48 h of growth, with and without IPTG induction

We used a combination of methods to study the response of *Synechocystis* to the two heterologous pathways. Both the dhurrin (DH) and the manoyl oxide (MO) producing strains exhibited slower growth rates (Fig. 2a). During the 1-day period, the control strain had an average doubling time of 11.5 h, while the DH and MO strains had doubling times of 14.7 and 12.7 h respectively. The dhurrin strain displays a more acute growth reduction after the IPTG induction. This inverse relationship between growth and product formation for both the dhurrin and manoyl oxide strains is not surprising and has been observed previously, e.g. [9, 11, 26, 27] since the organism allocates fixed carbon and reducing power towards the new metabolite—resources that would otherwise be available for biomass accumulation.

As previously reported [11], the DH strain secretes dhurrin to the culture medium reaching a concentration of 4.2 mg/L after 2 days of growth (the product yield is the same as reported in [11] at the same time point). The newly-engineered MO strain produces the heterologous diterpenoid manoyl oxide, reaching titers of 2 mg/L (~0.98 mg/g dry cell weight) of 13*R*-manoyl oxide after 2 days of growth (Fig. 2b). Normalized to cellular biomass, the manoyl oxide productivity increased more than twofold, compared to the previously reported 0.45 mg/g dry cell weight after 4 days of growth [10]. This is probably due to the different expression approach: in the current work a stronger promoter in a shuttle vector, as well as codon-optimized genes are used and in addition to the fact that the liquid cultures were supplemented with 3% CO_2_, which allows them to grow faster.

### Identification of correlated reactions

**Fig. 3:**
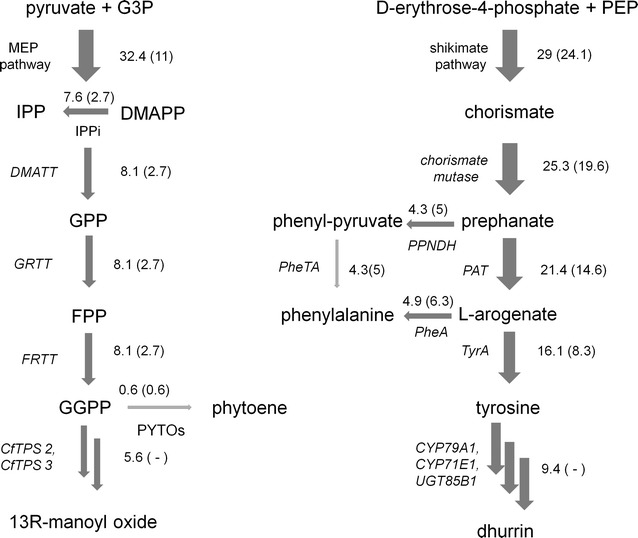
Predicted average fluxes of the biosynthetic pathways towards manoyl oxide (*left*) and dhurrin (*right*). The values were determined by random sampling (μmol product/gDW/h) in the presence and the absence of the heterologous biosynthetic pathway (*values outside and inside the brackets*, respectively). *G3P* glyceraldehyde 3-phosphate, *IPP* isopentenyl diphosphate, *DMAPP* dimethylallyl diphosphate, *GPP* geranyl diphosphate, *FPP* farnesyl diphosphate, *GGPP* geranylgeranyl diphosphate, *PEP* phosphoenolpyruvate

The dhurrin and the manoyl oxide biosynthesis initiate from tyrosine and GGPP, respectively. The consumption of these metabolites can potentially lead to imbalances in the cellular metabolism, while affecting reactions and pathways that are not directly linked to the heterologous reactions. Manoyl oxide derives form geranylgeranyl diphosphate (GGPP), produced in cyanobacteria via the methylerythritol phosphate (MEP) pathway (Fig. 3). Interactions between reactions in the metabolic networks were analyzed in terms of correlation coefficients calculated from the randomly sampled flux values (Additional file 2: Table S6). The metabolic reactions that displayed the strongest correlation with manoyl oxide productivity are listed in Additional file 1: Table S2. They are mainly reactions involved in forming MEP and GGPP. The isopentenyl diphosphate (IPP) isomerase reaction is computationally predicted to be negatively correlated with manoyl oxide secretion, highlighting the need of a balanced production of isopentenyl diphosphate (IPP) and dimethylallyl diphosphate (DMAPP). IPP and DMAPP react to form a geranyl diphosphate (GPP) molecule, which can be converted to GGPP with the addition of two IPP molecules (via farnesyl diphosphate) (Fig. 3). As a consequence, a proper equilibrium between IPP and DMAPP levels (namely 3:1) is required to maximize the GGPP formation. The enzyme that balances the two metabolite levels is the IPP isomerase, which reversibly converts IPP to DMAPP.

Dhurrin derives from tyrosine, which in turn originates from the shikimate pathway (Fig. 3). Correlation coefficients derived from random sampling show the dependence of dhurrin accumulation on flux in the shikimate pathway, as well as a connection with sugar catabolism and anabolism (glucose-1-phosphate uridylyltransferase activity correlates positively and phosphoglucomutase activity correlates negatively with dhurrin formation) (Additional file 1: Table S3).

Comparison of the predicted flux values in the modified strains with the predicted fluxes in the control strain (Fig. 3) shows that, in order to achieve the reported productivity for manoyl oxide, the flux from the MEP pathway requires an approximately threefold increase. The shikimate pathway also needs to increase its output by approximately 20%. It is also noteworthy that the increase in both the precursor pathways are predicted to be such as to roughly cover the extra demand posed by the heterologous reaction, while not affecting severely the fluxes of the branching pathways (Fig. 3).

### Effects on amino acids, carotenoids, and oxygen evolution

**Fig. 4:**
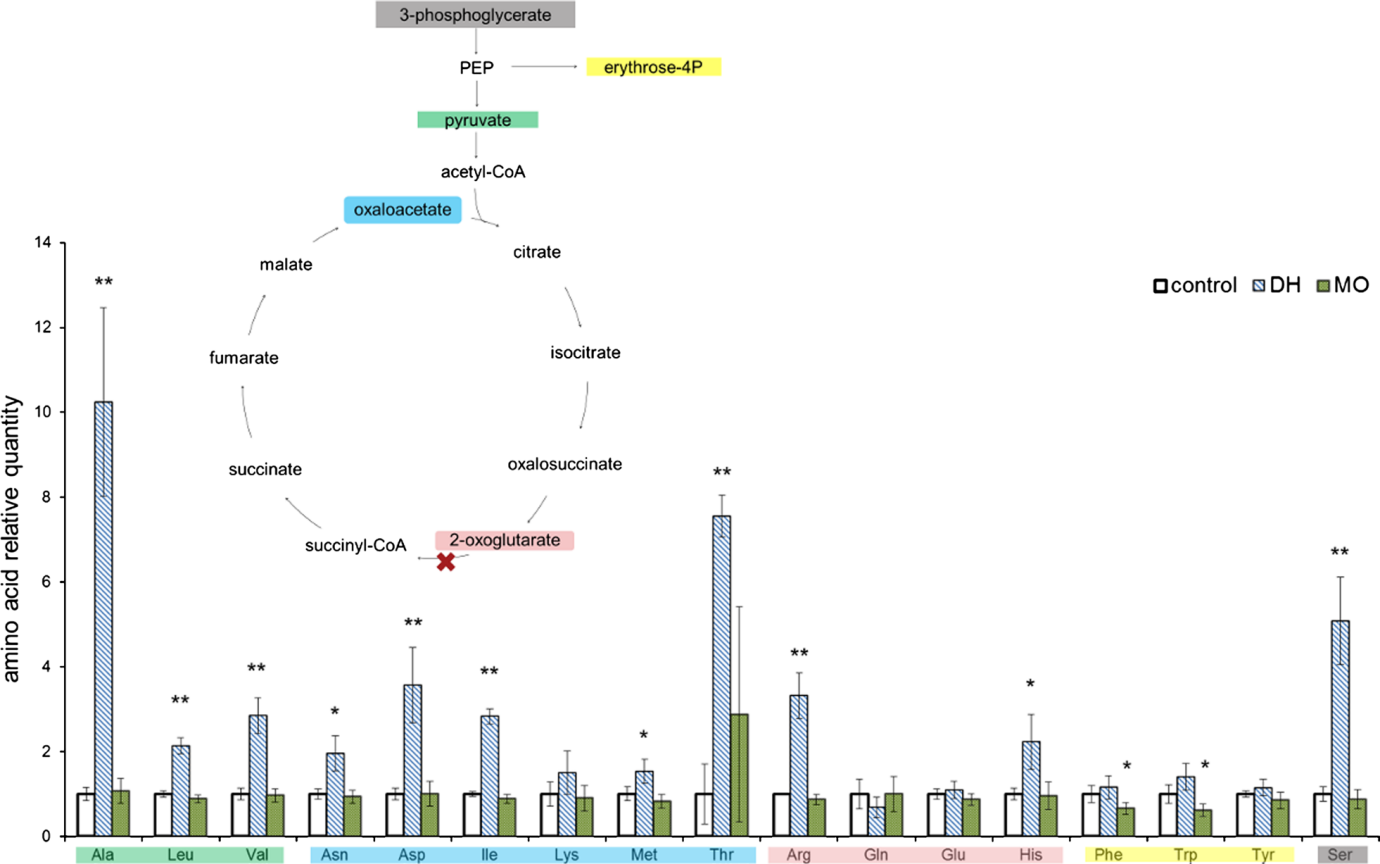
Amino acids relative quantity in the dhurrin (DH) and 13*R*-manoyl oxide (MO) producing strains. The significance of the differences between the control and the producer strains is indicated as *one star* (95% confidence) or *two stars* (99.5% confidence). The insert displays a part of the central carbon metabolism, where the precursors of the different groups of amino acid are colored in accordance with the corresponding colors of the amino acid labels in the *bar graph*. Data given as mean ± SD, N = 3

The role of aromatic amino acids in dhurrin formation and the computational predictions described above prompted us to determine the amino acid concentration of the both the manoyl oxide and dhurrin strains and compare these to the empty vector control (Additional file 1: Table S4). Among the 17 amino acids analyzed in the dhurrin strain we observed that the phenylalanine, tryptophan, and tyrosine concentrations were similar to the control (Fig. 4). However, most of the other amino acids accumulated in a much higher concentrations. In contrast, the manoyl oxide-producing strain did not display any dramatic difference in the amino acid content when compared to the empty vector control. The minor decrease in phenylalanine and tryptophan could be due to the overall decrease in growth (Fig. 2a).

These data suggest the presence of a response or regulatory mechanism that causes this non-predicted amino acid variation in the dhurrin strain. One could consider a scenario where there is an increase in the breakdown of storage carbohydrates. In the experiments, the strains were grown in high CO_2_ concentration, therefore they should have accumulated a large amount of glycogen [28]. After IPTG induction, the increased consumption of tyrosine for dhurrin biosynthesis will lead to increased erythrose-4P and phosphoenolpyruvate consumption, both of which participate in the central carbon metabolism (Fig. 4 insert). If the response to this perturbation is an increase in glycolysis, this could replenish the carbon pool. Consequently, the amino acid precursors would accumulate, leading to the increase in amino acids that was observed. Such effects have been reported previously, where the knockout of the glycogen synthase in *Synechococcus elongatus* PCC 7002 led to an over-accumulation of the TCA cycle metabolites [29], while an increased glycogen breakdown led to increased amino acid quantities [30]. We tested this hypothesis by quantifying the glycogen content in both the DH and the MO production strains and compared it to the empty vector control. Our results did not demonstrate any significant difference between the three strains in the glycogen content (Additional file 1: Figure S1), making a mechanism based on storage carbohydrate breakdown less likely. A potential reason for this could be that the sudden decrease in growth rate following IPTG induction—which is more pronounced than what we would expect solely by the redirection of metabolic resources towards dhurrin—causes an imbalance in carbon homeostasis, thereby perturbing the metabolic profile of the organism.

**Fig. 5:**
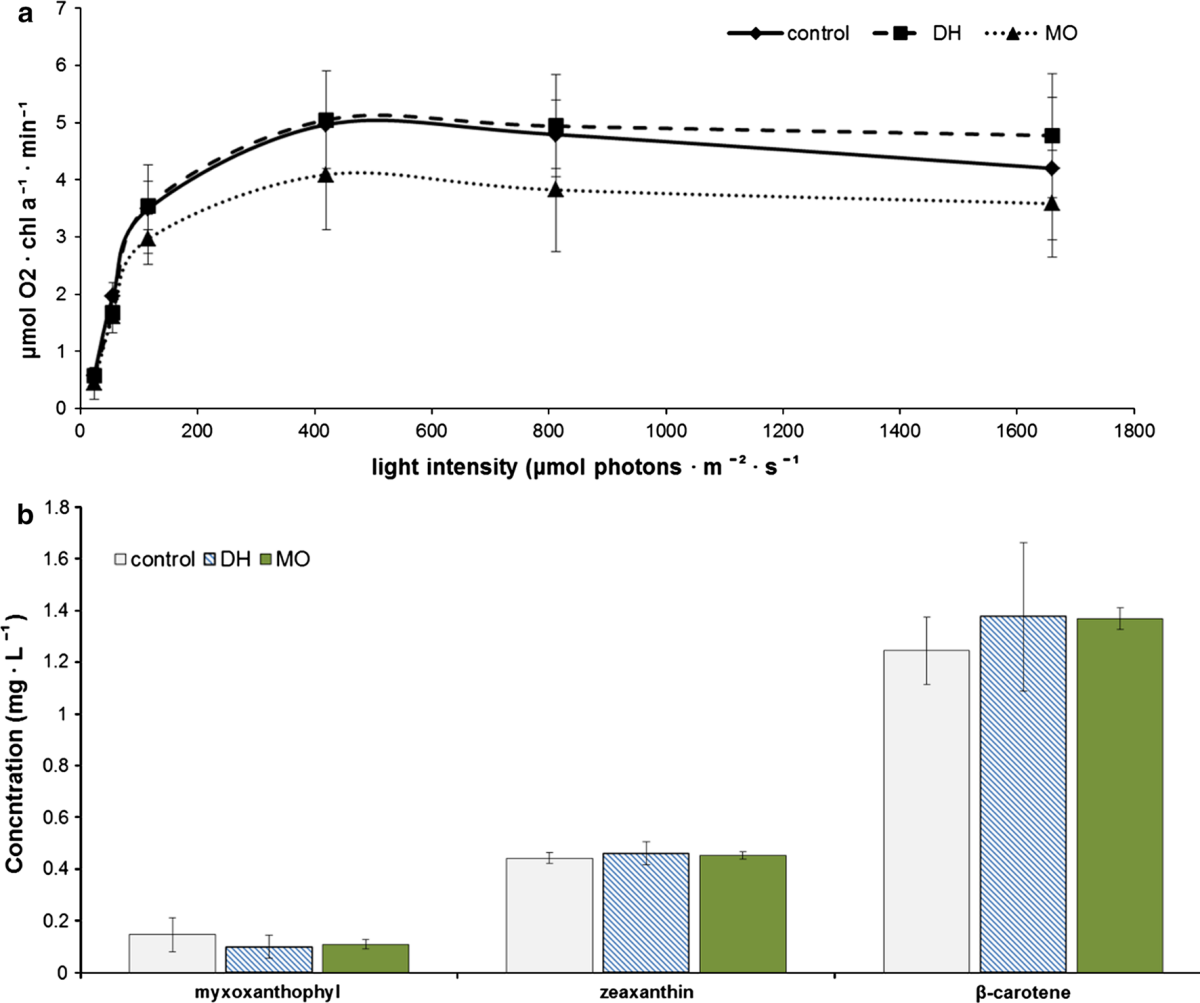
Oxygen evolution and carotenoid content in the dhurrin (DH) and 13*R*-manoyl oxide (MO) producing strains. **a** Oxygen evolution of the three strains, normalized to chlorophyll a content. **b** The three most abundant carotenoids were quantified with HPLC. Data given as mean ± SD, N = 3

Cyanobacteria rely on photosynthesis as an energy generator, and a variation in photosynthetic activity would affect many cellular and metabolic functions. Moreover, a significant upregulation of photosynthesis could explain a generic increase in the TCA cycle intermediates, and consequently an increase in amino acid levels. The amount of oxygen evolved by the cells provides an indication of differences in this aspect. Oxygen evolution was not found to be significantly affected when comparing the dhurrin-producer with the control (Fig. 5a). The manoyl oxide strain evolved slightly less oxygen; however, the differences were not significant and therefore the architecture and functionality of the photosynthetic apparatus is likely unaffected. The same small decrease in O_2_ evolution was observed in *Synechococcus* PCC 7492 during the production of the monoterpene limonene [31].

Carotenoids share the same precursor—GGPP—with manoyl oxide and we investigated whether the new competing pathway affect the pigmentation. We quantified the major photosynthetic pigments using extraction followed by HPLC analysis, and we see that indeed the cells were not affected in this aspect (Fig. 5b). The flux towards carotenoids was generally small, as indicated by the in silico flux analysis, and it was predicted to remain steady in the presence of the manoyl oxide biosynthetic pathway (Fig. 3).

## Discussion

The amount of fixed electrons directed towards dhurrin and manoyl oxide was 0.24 and 0.28% of the total fixed electrons respectively, determined by the ratio of the product titers and the biomass, as described in [2]. These numbers are small and appear to be well within the buffering capacity of the photosynthetic regulatory mechanisms. In support of this view, previous work [11, 32, 33], together with results presented here, indicate that the expression of cytochrome P450s does not drain a significant number of electrons out of the photosynthetic electron transfer chain. Dhurrin and manoyl oxide, being the product of long and complex metabolic routes, require a large amount of fixed carbons and subsequently harvested photons, as compared to simpler products, such as ethanol and succinic acid. Fixed carbon and its proper channeling appear to be the main restrictions for increased productivity, and not electron redirection, due to the versatility of the redox power distribution proteins [34].

*Synechocystis* displays a remarkable robustness, and is very recalcitrant to extended metabolic manipulations. Despite the fact that it is relatively straightforward to express a heterologous pathway and fine-tune expression levels and fluxes within the pathway itself, there are very few examples in the literature where high production titers have been achieved. Moreover, there is a lack of specific knowledge about how cyanobacteria regulate their metabolism. Cyanobacteria tend to compensate for large perturbations, and pinpointing the way they do that is crucial for successful genetic engineering. System biology approaches, namely—omics work coupled with computational methodology, can facilitate such an effort by locating connected metabolic pathways, identifying response mechanisms and off-target effects, and designing further improvement strategies [35]. Metabolic modelling and targeted metabolomics can quickly give valuable information on plausible effects of a given genetic modification, before more elaborate and resource-consuming methodologies, such as RNA sequencing and global metabolomics, are applied.

In this work we combined experimental and computational methods to understand how *Synechocystis* responds to the production of a diterpenoid and a cyanogenic glucoside. For both metabolites, the importance of channeling resources towards the biosynthesis of the precursor molecules is apparent, especially within the robust cyanobacterial metabolism. For dhurrin, there is a correlation with the catabolic reactions, while for the tyrosine derivatives resveratrol, *p*-coumaric acid, and caffeic acid, deletion of competing pathways, scaffolding, and expression optimization have previously been demonstrated efficient to increase production [36, 37]. Manoyl oxide production relies more on the balance between the different terpenoid metabolism branches and the efficient channeling through the MEP pathway. The overexpression of key enzymes of the GGPP biosynthesis has previously led to an increase of diterpenoid production in *Nicotiana benthamiana* leaves [15, 38], however this strategy was not successful when applied in *Synechocystis* [10]. Nevertheless, the careful study of the MEP pathway, the identification of the metabolic bottlenecks, and the optimization of fluxes via the screening of enzyme variants and enzyme fusions led to the high production of isoprene and limonene titers in *Synechococcus* PCC 7492 [9, 31]. Such a strategy can be generally applied to increase terpenoid production in cyanobacteria.

## Conclusions

We here present an example of how strain engineering approaches can be coupled to ‘traditional’ metabolic engineering with systems biology, resulting in better understanding of the organism as a system and potentially in novel and more efficient manipulation strategies. The insights acquired are applicable to a large number of other compounds that share biosynthetic routes and precursor molecules. We propose that the combination of metabolic modelling and metabolite analysis is an approach that can be adjusted to the production of any heterologous compound, forming a frame for understanding of the cyanobacterial cell as a microbial cell factory.

## Additional files

**Additional file 1: Table S1.** Reactions added to version 1.1 of the iJN678 model. **Table S2.** Reactions most strongly correlated with manoyl oxide production as determined by random sampling. **Table S3.** Reactions most strongly correlated with dhurrin production as determined by random sampling. **Table S4.** Amino acid concentrations (μmol/mL/OD730) of the control, dhurrin, and 13-*R* manoyl oxide producing strains. **Table S5.** Constraints applied in the three metabolic models used in this work. **Figure S1.** Glycogen content in the dhurrin (DH) and 13*R*-manoyl oxide (MO) producing strains compared to control (empty).

**Additional file 2: Table S6.** Average fluxes of reactions models, determined with random sampling.

**Additional file 3.** Updated metabolic models.

## Abbreviations

G3P: glyceraldehyde 3-phosphate
IPP: isopentenyl diphosphate
DMAPP: dimethylallyl diphosphate
GPP: geranyl diphosphate
FPP: farnesyl diphosphate
GGPP: geranylgeranyl diphosphate
PEP: phosphoenolpyruvate
DH: dhurrin
MO: 13*R*-manoyl oxide
MEP: methylerythritol phosphate pathway
IPTG: isopropyl β-d-1-thiogalactopyranoside
GC–MS: chromatography–mass spectrometry
HPLC: high performance liquid chromatography
