# Supplementary material for: Responses of Synechocystis sp. PCC 6803 to heterologous biosynthetic pathways
Source: Microb Cell Fact. 2017 Aug 15;16:140. doi: 10.1186/s12934-017-0757-y (PMC5556357; doi:10.1186/s12934-017-0757-y)
Supplement: Supplementary file 1 — Additional file 1: Table S1. Reactions added to version 1.1 of the iJN678 model. Table S2. Reactions most strongly correlated with manoyl oxide production as determined by random sampling. Table S3. Reactions most strongly correlated with dhurrin production as determined by random sampling. Table S4. Amino acid concentrations (μmol/mL/OD730) of the control, dhurrin, and 13-R manoyl oxide producing strains. Table S5. Constraints applied in the three metabolic models used in this work. Figure S1. Glycogen content in the dhurrin (DH) and 13R-manoyl oxide (MO) producing strains compared to control (empty). [file 12934_2017_757_MOESM1_ESM.doc]

# Supplemental material for Responses of *Synechocystis* sp. PCC 6803 to heterologous biosynthetic pathways

**Konstantinos Vavitsas**

Email: [kva@plen.ku.dk](mailto:kva@plen.ku.dk) Address: Copenhagen Plant Science Centre, Department of Plant and Environmental Sciences, University of Copenhagen, Thorvaldsensvej 40, 1871, Frederiksberg C, Denmark

**Emil Østergaard Rue**

Email: [kzw110@alumni.ku.dk](mailto:kzw110@alumni.ku.dk) Address: Copenhagen Plant Science Centre, Department of Plant and Environmental Sciences, University of Copenhagen, Thorvaldsensvej 40, 1871, Frederiksberg C, Denmark

**Lára Kristín Stefánsdóttir**

Email: [laramk90@gmail.com](mailto:laramk90@gmail.com) Address: Center for Systems Biology, University of Iceland, Sturlugata 8, 101 Reykjavik, Iceland

**Thiyagarajan Gnanasekaran**

Email: [gnanasek@insa-toulouse.fr](mailto:gnanasek@insa-toulouse.fr) Address: Copenhagen Plant Science Centre, Department of Plant and Environmental Sciences, University of Copenhagen, Thorvaldsensvej 40, 1871, Frederiksberg C, Denmark. Present address: ISBP - INSA de Toulouse, avenue de Rangueil, 31077 Toulouse, France

**Andreas Blennow**

Email: [abl@plen.ku.dk](mailto:abl@plen.ku.dk) Address: Copenhagen Plant Science Centre, Department of Plant and Environmental Sciences, and Center for Synthetic Biology ‘bioSYNergy’University of Copenhagen, Thorvaldsensvej 40, 1871, Frederiksberg C, Denmark

**Christoph Crocoll**

Email: [chcr@plen.ku.dk](mailto:chcr@plen.ku.dk) Address: Copenhagen Plant Science Centre and DynaMo Center, Department of Plant and Environmental Sciences, University of Copenhagen, Thorvaldsensvej 40, 1871, Frederiksberg C, Denmark

**Steinn Gudmundsson**

Email: [steinng@hi.is](mailto:steinng@hi.is) Address: Center for Systems Biology, University of Iceland, Sturlugata 8, 101 Reykjavik, Iceland

**Poul Erik Jensen (corresponding author)**

Email: [peje@plen.ku.dk](mailto:peje@plen.ku.dk) Address: Copenhagen Plant Science Centre, Department of Plant and Environmental Sciences, University of Copenhagen, Thorvaldsensvej 40, 1871, Frederiksberg C, Denmark

**Table S1**: **Reactions added to version 1.1 of the iJN678 model.** For the metabolite nomenclature refer to the documentation provided in the supplement of [1].

| **Name** | **Formula** | **Comment** | | **Reference** | |  |
| --- | --- | --- | --- | --- | --- | --- |
| SSDH | akg[c] + h[c] -> sucsal[c] + co2[c] | 2-oxoglutarate decarboxylase | | [2] | |  |
| FPK | f6p[c] + pi[c] -> actp[c] + e4p[c] + h2o[c] | phosphoketolase pathway | | [3] | |  |
| PSTA | 3php[c] + glu-L[c] -> 3ps[c] + akg[c] | phosphoserine pathway | | [4] | |  |
| PSP | 3ps[c] + h2o[c] -> ser-L[c] + pi[c] | phosphoserine pathway | | [4] | |  |
| GDH | glc-D[c] + nadp[c] + h2o[c] -> glcn[c] + nadph[c] + 2 h[c] | glucose dehydrogenase | | [5] | |  |
| GNK | atp[c] + glcn[c] -> 6pgc[c] + adp[c] + h[c] | gluconate kinase | | [5] | |  |
| EDD | 6pgc[c] -> 2ddg6p[c] + h2o[c] | phosphogluconate dehydratase | | [5] | |  |
| EDA | 2ddg6p[c] -> g3p[c] + pyr[c] | 2-dehydro-3-deoxy-phosphogluconate aldolase | | [5] | |  |
| PAT1 | pphn[c] + asp-L[c] + h[c] -> aro-L[c] + oaa[c] | prephenate transamination (aspartic donor) | | [6] | |  |
| PAT2 | pphn[c] + glu-L[c] + h[c] -> aro-L[c] + akg[c] | prephenate transamination (glutamate donor) | | [6] | |  |
| PHEA | aro-L[c] -> phe-L[c] + h2o[c] + co2[c] | Arogenate dehydratase to tyrosine | | [6] | |  |
| TYRA | aro-L[c] + nadp[c] -> h[c] + tyr-L[c] + nadph[c] + co2[c] | Arogenate dehydratase to phenylalanine | | [6] | |  |
| **Dhurrin biosynthesis reactions** | |  | |  |  | |
| **Name** | **Formula** | |  | | | |
| CYP79A1a | tyr-L[c] + o2[c] + fdxr-2_2[c] + 2 h[c] -> nhl-tyr[c] + fdxo-2_2[c] + h2o[c] | | | | |  |
| CYP79A1b | nhl-tyr[c] + o2[c] + fdxr-2_2[c] + 2 h[c] -> ndhl-tyr[c] + fdxo-2_2[c] + h2o[c] | | | | |  |
| CYP79A1c | ndhl-tyr[c] -> z4hpao[c] + co2[c] + h2o[c] | | | | |  |
| CYP71E1 | z4hpao[c] + fdxr-2_2[c] + 2 h[c] + o2[c] -> p-hmn[c] + fdxo-2_2[c] + 2 h2o[c] | | | | |  |
| UGT85B1 | udpg[c] + p-hmn[c] -> udp[c] + dhurrin[c] | | | | |  |
| **13R-manoyl oxide biosynthesis reactions** | | | | | |  |
| **Name** | **Formula** | | | | |  |
| CfTPS2 | ggdp[c] + h2o[c] -> c8odp[c] | | | | |  |
| CfTPS3 | c8odp[c] -> 13rmo[c] + h[c] + ppi[c] | | | | |  |

**Table S2: Reactions most strongly correlated with manoyl oxide production as determined by random sampling. The correlation factor takes values between -1 and 1 where 0 corresponds to no correlation. Reaction nomenclature is the same as in [1].**

| **Reaction name** | **Correlation factor** | **Pathway** |
| --- | --- | --- |
| CfTPS2 | 1.00 | Manoyl oxide biosynthesis |
| CfTPS1 | 1.00 | Manoyl oxide biosynthesis |
| FRTT | 1.00 | Sterol biosynthesis |
| CDPMEK | 1.00 | Terpenoid backbone biosynthesis |
| DXPS | 1.00 | Sterol biosynthesis |
| MEPCT | 1.00 | Sterol biosynthesis |
| DXPRIi | 1.00 | Sterol biosynthesis |
| MECDPS | 1.00 | Terpenoid backbone biosynthesis |
| MECDPDH_syn | 1.00 | Terpenoid backbone biosynthesis |
| DMATT | 1.00 | Terpenoid backbone biosynthesis |
| GRTT | 1.00 | Sterol biosynthesis |
| IPDPS_syn | 0.33 | Terpenoid backbone biosynthesis |
| DMPPS_syn | 0.33 | Terpenoid backbone biosynthesis |
| EX_h(e) | -0.20 | Exchange reactions |
| Htex | 0.20 | Transport |
| IPDDI | -0.17 | Sterol biosynthesis |
| NO3tex | -0.12 | Transport |
| EX_no3(e) | 0.12 | Exchange reactions |
| NOR_syn | -0.12 | Nitrogen metabolism |
| NAR_syn | -0.12 | Nitrogen metabolism |
| NO3abcpp | -0.12 | Transport |
| MALtpp | 0.11 | Transport |
| EX_mal-L(e) | -0.11 | Exchange reactions |
| MALtex | 0.11 | Transport |
| EX_cit(e) | -0.11 | Exchange reactions |
| CITtpp | 0.11 | Transport |
| CITtex | 0.11 | Transport |
| PGPP1836_9_12 | -0.10 | Fatty acid biosynthesis |
| ADMDC | -0.10 | Arginine and proline metabolism |
| MTRI | -0.10 | Sulfur Cysteine and methionine metabolism |

**Table S3: Reactions most strongly correlated with dhurrin production as determined by random sampling.** The correlation factor takes values between -1 and 1 where 0 corresponds to no correlation. Reaction nomenclature is the same as in [1].

| **Reaction name** | **Correlation factor** | **Pathway** |
| --- | --- | --- |
| CYP79A1c | 1.00 | Dhurrin biosynthesis |
| UGT85B1 | 1.00 | Dhurrin biosynthesis |
| CYP71E1 | 1.00 | Dhurrin biosynthesis |
| CYP79A1b | 1.00 | Dhurrin biosynthesis |
| TYRA | 1.00 | Dhurrin biosynthesis |
| GALU | 1.00 | Starch and sucrose metabolism |
| CHORM | 0.99 | Phenylalanine tyrosine and tryptophan biosynthesis |
| PGMT | -0.99 | Glycolysis/Gluconeogenesis |
| CHORS | 0.99 | Phenylalanine tyrosine and tryptophan biosynthesis |
| SHK3D | 0.99 | Phenylalanine tyrosine and tryptophan biosynthesis |
| SHKK | 0.99 | Phenylalanine tyrosine and tryptophan biosynthesis |
| DDPA | 0.99 | Phenylalanine tyrosine and tryptophan biosynthesis |
| DHQTi | 0.99 | Phenylalanine tyrosine and tryptophan biosynthesis |
| PSCVTi | 0.99 | Phenylalanine tyrosine and tryptophan biosynthesis |
| DHQS | 0.99 | Phenylalanine tyrosine and tryptophan biosynthesis |
| Htex | 0.42 | Transport |
| EX_h(e) | -0.42 | Exchange reactions |
| PAT2 | 0.11 | Phenylalanine tyrosine and tryptophan biosynthesis |
| DASYN1846_9_12_15 | -0.11 | Fatty acid biosynthesis |
| PGPP1846_9_12_15 | -0.11 | Fatty acid biosynthesis |
| SQD2_184_6_9_12_15 | -0.11 | Sulfolipid Biosynthesis |
| PC8XM | -0.11 | Porphyrin and chlorophyll metabolism |
| GLUCYS | -0.11 | Glutamate metabolism |
| PC6YM | -0.11 | Porphyrin and chlorophyll metabolism |
| GTHS | -0.11 | Glutamate metabolism |
| THRPS | -0.11 | Porphyrin and chlorophyll metabolism |
| DASYN161 | -0.11 | Fatty acid biosynthesis |
| ACBIPGT | -0.11 | Porphyrin and chlorophyll metabolism |
| PPK2r | 0.11 | Oxidative phosphorylation |

# Table S4: Amino acid concentrations (μmol mL^-1^ OD730^-1^) of the control, dhurrin, and 13-R manoyl oxide producing strains. Data given as mean ± SD, N=3

| **Amino acid** | **Ala** | **Arg** | **Asn** | **Asp** | **Gln** | **Glu** | **His** | **Ile** | **Leu** | **Lys** | **Met** | **Phe** | **Ser** | **Thr** | **Trp** | **Tyr** | **Val** |
| --- | --- | --- | --- | --- | --- | --- | --- | --- | --- | --- | --- | --- | --- | --- | --- | --- | --- |
| Control | 2.35 ±0.36 | 2.30 ± 0.07 | 0.35 ±0.04 | 1.36 ±0.18 | 43.60 ±15.24 | 8.58 ±1.09 | 0.30 ±0.04 | 0.42 ±0.03 | 0.83 ±0.06 | 2.57 ±0.75 | 0.17 ±0.03 | 0.75 ±0.16 | 1.90 ±0.36 | 0.23 ±0.16 | 0.04 ±0.01 | 0.26 ±0.02 | 0.72 ±0.10 |
| Dhurrin strain | 24.05 ±3.6 | 7.63 ± 1.22 | 0.69 ±0.12 | 4.85 ±1.01 | 29.78 ±2.359 | 9.40 ±1.23 | 0.67 ±0.17 | 1.20 ±0.01 | 1.79 ±0.09 | 3.86 ±0.68 | 0.26 ±0.02 | 0.86 ±0.09 | 9.66 ±0.98 | 1.73 ±0.86 | 0.05 ±0.01 | 0.30 ±0.04 | 2.04 ± 0.12 |
| Manoyl oxide strain | 2.51 ±0.57 | 2.00 ±0.26 | 0.33 ±0.04 | 1.36 ±0.35 | 43.73 ±9.63 | 7.45 ±0.72 | 0.29 ±0.09 | 0.37 ±0.04 | 0.74 ±0.05 | 2.31 ±0.39 | 0.14 ±0.02 | 0.49 ±0.02 | 1.67 ±0.34 | 0.66 ±0.34 | 0.02 ±0.01 | 0.22 ±0.05 | 0.69 ±0.06 |

# Table S5: Constraints applied in the three metabolic models used in this work.

|  | **Control** |  |  | **Dhurrin** |  |  | **Manoyl oxide** | |
| --- | --- | --- | --- | --- | --- | --- | --- | --- |
| Rxn ID | lower | upper |  | lower | upper |  | lower | upper |
|  | mmol/gDW/h | mmol/gDW/h | | mmol/gDW/h | mmol/gDW/h | | mmol/gDW/h | mmol/gDW/h |
| CfTPS1 | 0 | 0 |  | 0 | 0 |  | -1000 | 1000 |
| CfTPS2 | 0 | 0 |  | 0 | 0 |  | -1000 | 1000 |
| CYP79A1a | 0 | 0 |  | -1000 | 1000 |  | 0 | 0 |
| CYP79A1b | 0 | 0 |  | -1000 | 1000 |  | 0 | 0 |
| CYP79A1c | 0 | 0 |  | -1000 | 1000 |  | 0 | 0 |
| CYP71E1 | 0 | 0 |  | -1000 | 1000 |  | 0 | 0 |
| UGT85B1 | 0 | 0 |  | -1000 | 1000 |  | 0 | 0 |
| EX_h2o(e) | -1000 | 1000 |  | -1000 | 1000 |  | -1000 | 1000 |
| EX_o2(e) | -1000 | 1000 |  | -1000 | 1000 |  | -1000 | 1000 |
| EX_hco3(e) | -3,7 | 1000 |  | -3,7 | 1000 |  | -3,7 | 1000 |
| EX_no3(e) | -1000 | 1000 |  | -1000 | 1000 |  | -1000 | 1000 |
| EX_k(e) | -1000 | 1000 |  | -1000 | 1000 |  | -1000 | 1000 |
| EX_so4(e) | -1000 | 1000 |  | -1000 | 1000 |  | -1000 | 1000 |
| EX_pi(e) | -1000 | 1000 |  | -1000 | 1000 |  | -1000 | 1000 |
| EX_mobd(e) | -1000 | 1000 |  | -1000 | 1000 |  | -1000 | 1000 |
| EX_fe3(e) | -1000 | 1000 |  | -1000 | 1000 |  | -1000 | 1000 |
| EX_fe2(e) | -1000 | 1000 |  | -1000 | 1000 |  | -1000 | 1000 |
| EX_na1(e) | -1000 | 1000 |  | -1000 | 1000 |  | -1000 | 1000 |
| EX_mn2(e) | -1000 | 1000 |  | -1000 | 1000 |  | -1000 | 1000 |
| EX_mg2(e) | -1000 | 1000 |  | -1000 | 1000 |  | -1000 | 1000 |
| EX_cu2(e) | -1000 | 1000 |  | -1000 | 1000 |  | -1000 | 1000 |
| EX_zn2(e) | -1000 | 1000 |  | -1000 | 1000 |  | -1000 | 1000 |
| EX_cobalt2(e) | -1000 | 1000 |  | -1000 | 1000 |  | -1000 | 1000 |
| EX_ni2(e) | -1000 | 1000 |  | -1000 | 1000 |  | -1000 | 1000 |
| EX_ca2(e) | -1000 | 1000 |  | -1000 | 1000 |  | -1000 | 1000 |
| EX_photon(e) | -100 | -100 |  | -100 | -100 |  | -100 | -100 |
| EX_h(e) | -1000 | 1000 |  | -1000 | 1000 |  | -1000 | 1000 |
| Sink_precyanphy | -1000 | 1000 |  | -1000 | 1000 |  | -1000 | 1000 |
| Sink_phbg | -1000 | 1000 |  | -1000 | 1000 |  | -1000 | 1000 |
| Sink_rdmbzi | -1000 | 1000 |  | -1000 | 1000 |  | -1000 | 1000 |
| Sink_dna | -1000 | 1000 |  | -1000 | 1000 |  | -1000 | 1000 |
| DM_13rmo[c] | 0 | 0 |  | 0 | 0 |  | 0,0008 | 1000 |
| DM_dhurrin[c] | 0 | 0 |  | 0,0023 | 1000 |  | 0 | 0 |
| Ec_biomass_SynAuto | 0,059 | 1000 |  | 0,047 | 1000 |  | 0,0544 | 1000 |

#
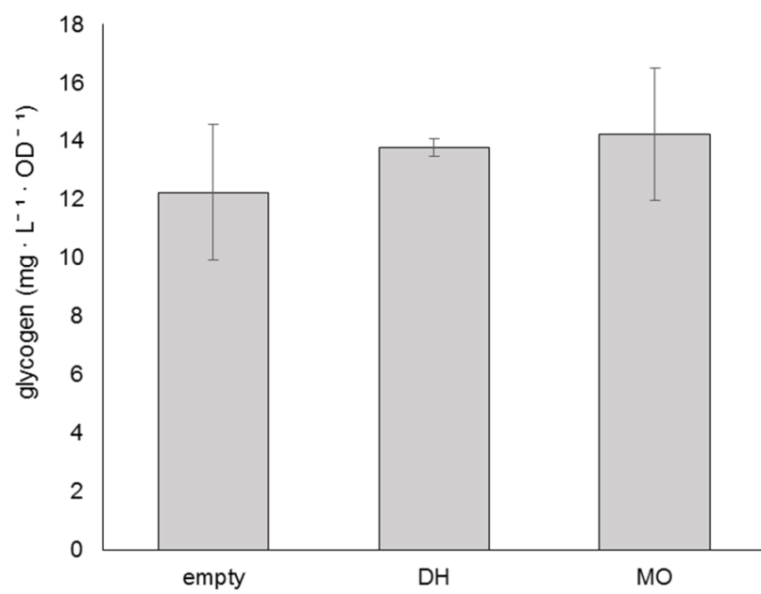
Fig. S1: Glycogen content in the dhurrin (DH) and 13R-manoyl oxide (MO) producing strains compared to control (empty). The differences observed between the strains were not found to be statistically significant (95% confidence level). Data given as mean ± SD, N=3.

# References

1. Nogales J, Gudmundsson S, Knight EM, Palsson BO, Thiele I. Detailing the optimality of photosynthesis in cyanobacteria through systems biology analysis. Proc. Natl. Acad. Sci. 2012;109:2678–83. A

2. Zhang S, Bryant DA. The Tricarboxylic Acid Cycle in Cyanobacteria. Science 2011;334:1551–3.

3. Xiong W, Lee T-C, Rommelfanger S, Gjersing E, Cano M, Maness P-C, et al. Phosphoketolase pathway contributes to carbon metabolism in cyanobacteria. Nat. Plants 2015;2:15187.

4. Klemke F, Baier A, Knoop H, Kern R, Jablonsky J, Beyer G, et al. Identification of the light-independent phosphoserine pathway as an additional source of serine in the cyanobacterium Synechocystis sp. PCC 6803. Microbiology 2015;161:1050–60.

5. Chen X, Schreiber K, Appel J, Makowka A, Fähnrich B, Roettger M, et al. The Entner–Doudoroff pathway is an overlooked glycolytic route in cyanobacteria and plants. Proc. Natl. Acad. Sci. 2016;113:5441–6. A

6. Bonner CA, Jensen RA, Gander JE, Keyhani NO. A core catalytic domain of the TyrA protein family: arogenate dehydrogenase from Synechocystis. Biochem. J. 2004;382:279–91.
